# Rural Resilience Through COVID-19

**DOI:** 10.1093/geroni/igab046.1617

**Published:** 2021-12-17

**Authors:** Shannon Freeman, Raven Weaver, Shannon Freeman

## Abstract

The effects of the COVID-19 pandemic have been felt globally affecting everyone, but have disproportionately harmed some of the most vulnerable and marginalized including individuals residing in rural and remote areas. The geographic isolation initially thought to protect rural and remote communities from the pandemic soon became a disadvantage, requiring individuals to navigate long-standing systemic barriers (e.g., lack of transportation issues, limited access to healthcare resources, and fragmented accessibility to vaccines), alongside the new challenges posed by COVID-19 restrictions to mitigate the spread of disease. The purpose of this symposium is to showcase examples of rural resiliency in the face of significant struggle. Taking a strength-based approach, the papers discuss efforts to identify healthy coping and positive aspects of physical distancing (Paper 1; Weaver), explore social support and psychological mindset (Paper 2; Fuller), inform successful strategies to pivot programming to remote coalition engagement for obesity prevention (Paper 3; Buys), implement a peer mentoring program to spur development of new strategies to build community resilience (Paper 4; Oh), and review elements of rurality that empower or exclude older people and the implications for a post-COVID world (Paper 5; Curreri). As we continue to uncover and learn about the short and long-term implications of living through the pandemic, these papers describe ways in which rural communities demonstrate resilience in the face of adversity. Our presenters will showcase a range of US and international perspectives and offer policy and program recommendations for building resilience in the longer term.

